# Characterization of Cell Cycle-Related Competing Endogenous RNAs Using Robust Rank Aggregation as Prognostic Biomarker in Lung Adenocarcinoma

**DOI:** 10.3389/fonc.2022.807367

**Published:** 2022-02-03

**Authors:** Yifei Yang, Shiqi Zhang, Li Guo

**Affiliations:** ^1^ Department of Bioinformatics, Smart Health Big Data Analysis and Location Services Engineering Lab of Jiangsu Province, School of Geographic and Biologic Information, Nanjing University of Posts and Telecommunications, Nanjing, China; ^2^ Department of Biology, Brandeis University, Waltham, MA, United States

**Keywords:** lung adenocarcinoma (LUAD), competing endogenous RNA (ceRNA), robust rank aggregation (RRA), cross-talk, cancer prognosis

## Abstract

Lung adenocarcinoma (LUAD), one of the most common pathological subtypes in lung cancer, has been of concern because it is the leading cause of cancer-related deaths. Due to its poor prognosis, to identify a prognostic biomarker, this study performed an integrative analysis to screen curial RNAs and discuss their cross-talks. The messenger RNA (mRNA) profiles were primarily screened using robust rank aggregation (RRA) through several datasets, and these deregulated genes showed important roles in multiple biological pathways, especially for cell cycle and oocyte meiosis. Then, 31 candidate genes were obtained *via* integrating 12 algorithms, and 16 hub genes (containing homologous genes) were further screened according to the potential prognostic values. These hub genes were used to search their regulators and biological-related microRNAs (miRNAs). In this way, 10 miRNAs were identified as candidate small RNAs associated with LUAD, and then miRNA-related long non-coding RNAs (lncRNAs) were further obtained. In-depth analysis showed that 4 hub mRNAs, 2 miRNAs, and 2 lncRNAs were potential crucial RNAs in the occurrence and development of cancer, and a competing endogenous RNA (ceRNA) network was then constructed. Finally, we identified CCNA2/MKI67/KIF11:miR-30a-5p:VPS9D1-AS1 axis-related cell cycle as a prognostic biomarker, which provided RNA cross-talks among mRNAs and non-coding RNAs (ncRNAs), especially at the multiple isomiR levels that further complicated the coding–non-coding RNA regulatory network. Our findings provide insight into complex cross-talks among diverse RNAs particularly involved in isomiRs, which will enrich our understanding of mRNA–ncRNA interactions in coding–non-coding RNA regulatory networks and their roles in tumorigenesis.

## Highlights

- The abnormal mRNA profiles in LUAD were primarily characterized using the RRA algorithm. The 16 potential hub genes were screened *via* PPI network and survival analysis, and some of them were identified as homologous members in the gene family.
- Related miRNAs were surveyed based on the 16 hub genes, and miRNA-associated lncRNAs were further screened. Then, 4 mRNAs, 2 miRNAs, and 2 lncRNAs were identified as key RNAs to construct a ceRNA network.
- Further in-depth analysis characterized CCNA2/MKI67/KIF11:miR-30a-5p:VPS9D1-AS1 axis-related cell cycle as a prognostic biomarker, and all of these RNAs were cancer-associated crucial genes.

## Introduction

Lung cancer, one of the most common fatal cancers, has been the leading cause of cancer-related deaths, with an increasing incidence worldwide (1). This cancer can be categorized into 2 major types, non-small cell lung cancer (NSCLC; ~85%) and small cell lung cancer (SCLC; ~15%). The former is further classified into three major subtypes according to histopathology and clinical features: lung adenocarcinoma (LUAD; ~40%), lung squamous cell carcinoma (LUSC; ~25%–30%), and large cell carcinoma (LCC; ~10%–15%). LUAD and LUSC are the most common pathological subtypes in lung cancer (2–4), and LUAD is specifically the most frequent subtype in never or light smokers (5). LUAD patients are mainly caused by a combination of multiple genetic and environmental factors (6). The prognosis of NSCLC patients is not optimistic, and the 5-year survival rate is less than 1% (7, 8), which is mainly attributed to regional or distant metastasis (9, 10). Patients often have little opportunity of receiving effective treatments because they lack specific clinical symptoms and therefore are diagnosed at a very late stage. Characterization of new cancer-specific diagnostic and prognostic biomarkers is quite necessary, which will greatly assist in timely diagnosis, prognosis, treatment selection, and guiding further clinical treatment.

In recent years, non-coding RNA (ncRNA), mainly including microRNA (miRNA), long ncRNA (lncRNA), and circular RNA (circRNA), has been widely studied as a class of important regulatory molecules, especially for their crucial roles in tumorigenesis (11–13). These ncRNAs have been of interest because of their potential roles as biomarkers for the diagnosis and prognosis of various cancers (14–16). The interactions with messenger RNAs (mRNAs), especially *via* competing endogenous RNAs (ceRNAs), indicate that ncRNAs and mRNAs can function as ceRNAs by competitively binding with miRNAs through sharing miRNA recognition elements to regulate their expression levels (17). Based on this hypothesis, relevant RNAs have been studied, particularly for their potential prognostic roles in tumorigenesis. For example, the circRNA hsa_circ_0072088, miRNAs (hsa-miR-532-3p and hsa-miR-942-5p), and mRNAs (IGF2BP3, MKI67, CD79A, and ABAT) may serve as prognostic markers in LUAD *via* a circRNA-mediated ceRNA network (18); LINC00324/miR-9-5p (miR-33b-5p)/GAB3 (IKZF1) may play a pivotal role in regulating TAM risk and prognosis in LUAD patients (19), and some studies focus on cancer-related lncRNAs to search crucial RNA interactions based on ceRNA networks (20, 21). These studies provide potential crucial gene interactions in tumorigenesis, which are quite necessary to reveal the detailed molecular mechanism of diverse cancers. However, it is not enough to present these interactions from these RNA levels, because the small regulatory RNA, miRNA, is not a single sequence but a series of multiple isomiRs (22–26). Do these small flexible isomiRs also contribute to RNA cross-talks and the occurrence and development of cancers? It is urgent to explore these interactions at the isomiR levels, which will help us understand the interesting cross-talks in the RNA world.

**Figure 1:**
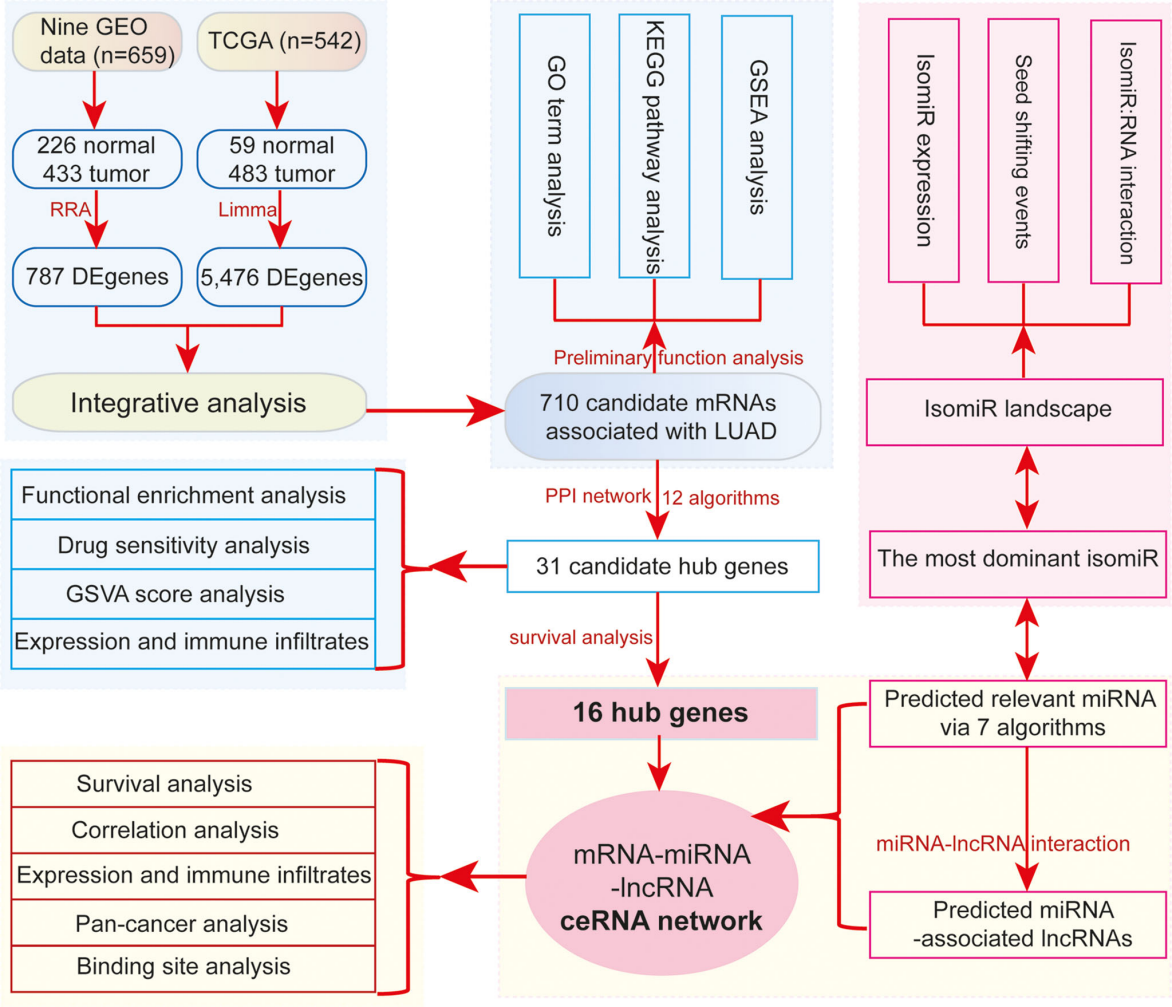
The main flowchart of the study. DE genes, deregulated genes.

In this study, to further understand the potential cross-talks among diverse RNAs in LUAD (**Figure 1**), we mainly discuss the interactions among ncRNAs and mRNAs, particularly from the isomiR level. Firstly, *via* an integrative analysis of several datasets, consistent deregulated genes are surveyed using robust rank aggregation (RRA) algorithm, and their functional implications are queried to understand the potential contributions in tumorigenesis. Secondly, protein–protein interaction (PPI) networks are used to screen potential hub genes associated with cancer through integrating multiple algorithms, and these hub genes are further screened by survival analysis. Thirdly, relevant miRNAs of these hub genes are obtained, and then these interacted miRNAs are used to survey related lncRNAs. Finally, based on the potential biological interactions, a ceRNA network is constructed, and involved RNAs are further analyzed to understand their expression correlations and potential roles in tumorigenesis, especially for the analysis at the isomiR level. Our study will provide insight into RNA cross-talks and more references for potential crucial RNAs associated with lung cancer, particularly focusing on coding–non-coding RNA interaction networks at the isomiR level. These findings will contribute to discovering the novel potential anticancer drug target in precision medicine.

## Materials and Methods

### Data Resource

In order to obtain deregulated mRNAs in LUAD, we obtained 659 samples (tumor, 433; normal, 226) from 9 datasets in the Gene Expression Omnibus (GEO; https://www.ncbi.nlm.nih.gov/geo/, GSE31210, GSE118370, GSE75037, GSE32863, GSE85716, GSE85841, GSE63459, GSE130779, and GSE148036) by GEOquery (27) and 542 (tumor, 483, normal, 59) samples from The Cancer Genome Atlas (TCGA; https://tcga-data.nci.nih.gov/tcga/) using the “TCGAbiolinks” package (http://doi.org/10.1093/nar/gkv1507) (28) (**Table S1**). High-throughput RNA sequencing data (including mRNA, lncRNA, and miRNA/isomiR) in diverse cancer types were also obtained from TCGA, which were mainly used to detect the detailed pan-cancer expression patterns of screened crucial genes in LUAD.

### Screening and Identification of Deregulated RNAs

The limma (29) was used to screen and identify deregulated RNAs in GEO and TCGA datasets using the Bioconductor packages. The common candidate cancer-associated mRNAs were firstly screened using R package RobustRankAggreg (30) in 9 GEO datasets, and candidate mRNAs were further analyzed with deregulated mRNA profiles from TCGA dataset. mRNAs with |log_2_FC| > 1 and padj < 0.05 were primarily identified as abnormally expressed genes.

### Functional Enrichment Analysis of Gene Sets

To understand the detailed functional implication of differentially expressed gene sets or screened specific genes, the Database for Annotation, Visualization and Integrated Discovery (DAVID) version 6.8 (31) and clusterProfiler (32) were used to perform functional analysis. Simultaneously, based on identified Kyoto Encyclopedia of Genes and Genomes (KEGG) pathways, z scores were estimated according to the following formula (33):


(1) $$z–score=\frac{\left(up-down\right)}{\sqrt{count}}$$

where the up and down indicate the numbers of upregulated and downregulated genes, respectively, and the count was the total number of involved deregulated genes.

Furthermore, to understand the detailed expression patterns of the screened genes, their expression distributions in KEGG pathways were also queried, and significantly enriched pathways were further presented using Pathview (34, 35). A p-value <0.05 was considered to have statistical significance.

### Screening and Identification of Potential Cancer-Associated Hub Genes

To survey the potential hub genes in LUAD, PPI networks were firstly constructed based on deregulated mRNA profiles using the STRING online database with default parameters (36). Networks were constructed using upregulated and downregulated genes. For the PPI network, the candidate key genes were firstly screened based on the potential modules using the CytoHubba plug-in in Cytoscape 3.7.2 (37). Then, we selected the top 10 node genes from 12 algorithms results (including Betweenness, BottleNeck, Closeness, ClusteringCoefficient, Degree, DMNC, EcCentricity, EPC, MCC, MNC, Radiality, and Stress) as candidate genes. Genes with degree scores <10 were excluded, and the remaining genes detected in more than 4 other algorithms were finally selected as candidate hub genes. We here used the PageRank algorithms to explore the hub genes from the significant-difference expression genes. As a method of evaluating the importance of nodes, the PageRank was also a useful algorithm to explore the relative topological importance, and the PageRank had been used to discover the herb’s relative importance and determine the core herbs (38).

For primarily screened hub genes, further analysis was performed to understand the potential role in tumorigenesis, mainly including drug sensitivity and correlations between hub genes and immune infiltrates (http://bioinfo.life.hust.edu.cn/web/GSCALite/) (39). Moreover, gene set variation analysis (GSVA) scores for hub gene sets were also estimated using GSCALite.

### Characterization of Potential Prognostic Values of Candidate Genes

It was necessary to query the potential prognostic values of the screened cancer-associated hub genes, which will help us to understand their roles in tumorigenesis. Then, survival analyses were used to estimate the correlations of the candidate genes (also including further screened candidate miRNAs and lncRNAs) with cancer prognoses. The clinical data, mainly including survival status, cancer stage and grade, survival time, and molecular subtype, were obtained from TCGA using the “TCGAbiolinks” package (28). The log-rank test was used to estimate the potential differences, and statistical significance was set at p < 0.05. Simultaneously, in order to obtain the integrated results to ensure the potential prognostic values of screened genes, prognostic results were also obtained from the GEPIA (40, 41) and StarBase (42, 43) databases.

### Screening and Identification of Relevant Cancer-Associated Non-Coding RNAs

Candidate hub mRNAs with potential prognostic values were firstly used to screen related miRNAs based on biological interactions because the small ncRNAs have been widely studied as a class of important regulators in gene expression. The miRNA:mRNA interactions were firstly collected from the StarBase database (42, 43), and those miRNAs remained as candidate-related miRNAs if they had opposite expression patterns with target mRNAs and had significant prognostic results. Here, due to the phenomenon of multiple isomiRs in the miRNA locus (22–26), we selected the most dominant isomiR as the classical miRNA to perform the relevant analysis. The detailed isomiR expression patterns were further queried for the final screened cancer-associated crucial miRNAs, because the multiple isomiRs may lead to perturbed coding–non-coding RNA regulatory network (44) that may also perturb the ceRNA network.

Next, based on the screened miRNAs that were crucial intermediate nodes correlating mRNAs and lncRNAs, miRNA-related deregulated lncRNAs were further surveyed from LncBase Predicted v.2 (45), and lncRNAs were identified if they had opposite expression patterns with miRNAs and had potential prognostic values in cancer prognosis.

### Construction of Competing Endogenous RNA Network to Screen Cancer-Associated Crucial RNAs

According to screened cancer-associated abnormal RNAs, mainly including hub genes, interacted miRNAs, and associated lncRNAs, a ceRNA network was constructed based on their regulatory relationships using the R package of “networkD3” (https://CRAN.R-project.org/package=networkD3). The primary constructed ceRNA network contained a series of mRNAs and ncRNAs, and then these related mRNA:miRNA and miRNA:lncRNA pairs were further queried for their expression relationships. A correlation analysis was used to estimate their expression correlations, and if the correlation coefficient was less than −0.20, p < 0.05, and the average expression level (log_2_TPM) was more than 10 (ensure the abundant enrichment level), further analysis of the genes remains to be performed.

### In-Depth Analysis for Screened Crucial RNAs

Moreover, although all of the above-screened associated genes were dominantly and abnormally expressed in tumor samples, and they also had significant correlations with cancer prognosis, it is necessary to further understand the expression patterns across diverse cancer types (46) that will help us assess the potential expression and function of genes in different tissues and tumorigenesis. Therefore, a pan-cancer analysis was used to track their expression patterns. Simultaneously, the binding events of diverse RNAs were visualized using DIANA (http://carolina.imis.athena-innovation.gr/diana_tools/web/index.php?r=site%2Findex) (47, 48), which could indicate the interactions among different RNAs in the ceRNA network. Furthermore, the screened crucial mRNAs were queried for the potential roles in immune infiltrates in LUAD (46), which would contribute to understanding the biological role of the hub genes.

### Statistical Analysis and Network Visualization

An unpaired t-test and the Wilcoxon rank-sum test were used to estimate differentially expressed genes for the unpaired samples. For interactions between related genes, especially among different RNAs, further network visualization was presented using Cytoscape 3.8.2 (37). A Pearson’s or Spearman’s correlation coefficient was estimated to assess expression relationships among different RNAs. All of these statistical analyses were performed using the R programming language (version 3.4.3), and Venn distributions were performed with a publicly available tool (http://bioinformatics.psb.ugent.be/webtools/Venn/).

## Results

### Messenger RNA Expression Profile in Lung Adenocarcinoma

**Figure 2:**
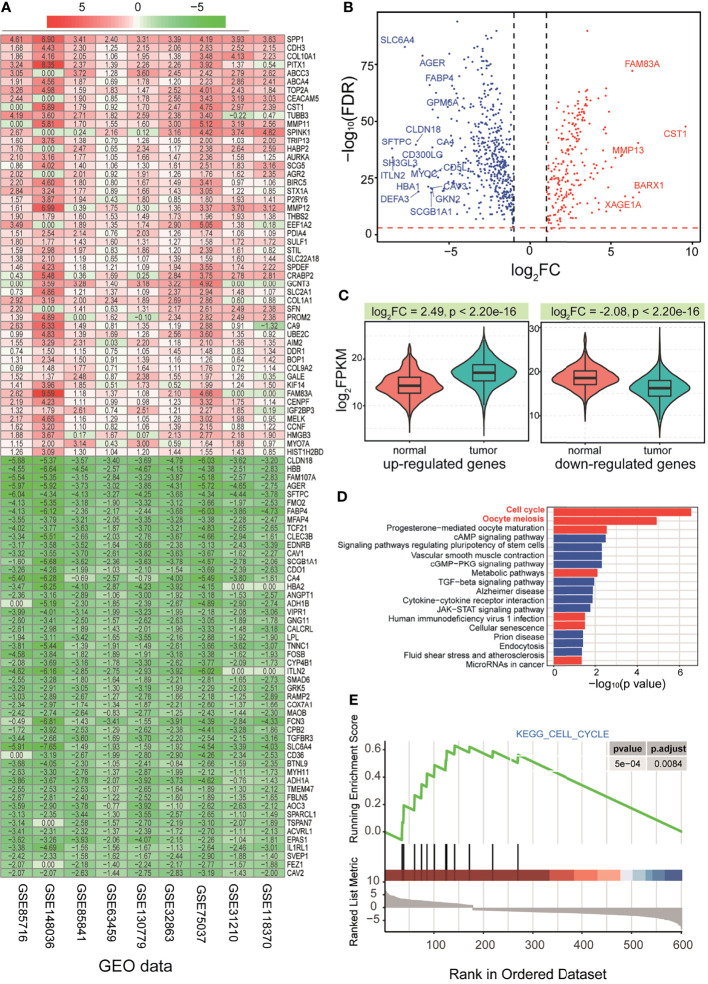
Screening candidate genes and functional analysis *via* an integrative analysis of multiple datasets. **(A)** A heatmap of distributions of RRA scores in 9 GEO datasets. **(B)** Expression distributions for screened 710 common genes *via* GEO and TCGA datasets. **(C)** Expression patterns (based on the median values of TPM) for all the screened up-deregulated and downregulated genes, and a p-value based on t-test is also presented. **(D)** Significant enriched KEGG pathways of screened deregulated genes. **(E)** GSEA in significant KEGG cell cycle pathways. RRA, robust rank aggregation; GEO, Gene Expression Omnibus; TCGA, The Cancer Genome Atlas; KEGG, Kyoto Encyclopedia of Genes and Genomes; GSEA, gene set enrichment analysis.

According to 9 GEO datasets (**Table S1** and **Figure 1**), the RRA algorithm was used to screen deregulated mRNAs, and a total of 787 abnormally expressed genes were obtained based on distributions of scores in the RRA algorithm (**Figures 2A** and **S1A**). Subsequently, 5,476 abnormally expressed genes were obtained from TCGA data (**Figure S1A**), and 710 genes (including 474 downregulated genes and 236 upregulated genes) with consistent expression patterns were collected as candidate genes to perform further analysis (**Figure 2B**). Some abnormal genes were reported with important roles in tumorigenesis. For example, upregulated CST1 can promote gastric cancer migration and invasion through activating the Wnt pathway (49), and CST1 also promotes cell proliferation, clone formation, and metastasis in breast cancer cells, indicating that CST1 is a novel potential prognostic biomarker and therapeutic target for breast cancer (50). The screened upregulated and downregulated genes were further queried for their expression patterns, respectively, and we found that both of them showed significant expression differences (**Figure 2C**, p = 2.20e−16 for the upregulated genes and p = 2.20e−16 for the downregulated genes). Most of them showed abundant expression distributions, indicating that these screened candidate genes were dominantly expressed in LUAD.

To understand whether these surveyed genes had a potential function, functional enrichment analysis was performed. Both upregulated and downregulated genes showed significant Gene Ontology (GO) terms (**Figures S1C, D**), indicating that these abnormal genes might contribute to multiple biological processes. These primarily screened genes were also enriched in several KEGG pathways, especially for cell cycle and oocyte meiosis pathways (**Figures 2D, E**, and **S2**). In the detailed pathways, many relevant genes were involved in deregulated expression patterns (**Figure S3A**), which may perturb the relevant pathways.

### Screening of the Potential Most Influential Genes in Protein–Protein Interaction Networks

**Figure 3:**
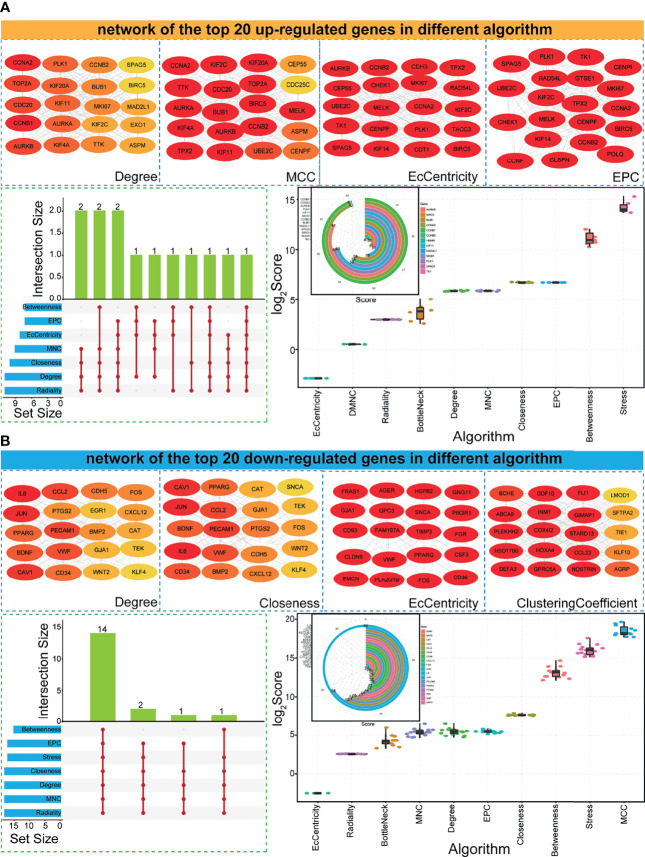
Potential hub genes *via* PPI networks based on different algorithms. **(A)** Examples of PPI networks using different algorithms (each network contains the top 20 upregulated genes). The darker the red background color of the gene, the higher the ranking of the gene. The gene distributions in different algorithms, and score distributions for surveyed genes in diverse algorithms. The score of degree is also presented for screened 13 hub genes. **(B)** Examples of PPI networks using different algorithms (each network contains the top 20 downregulated genes). The darker the red, the higher the ranking. The gene distributions in different algorithms, and score distributions for surveyed genes in diverse algorithms. The score of degree is also presented for 18 screened hub genes. PPI, protein–protein interaction.

Based on the obtained upregulated and downregulated gene sets, the PPI network was constructed. According to the primarily constructed complex networks, the potential hub genes were further screened using 12 different algorithms. Based on the top 20 genes in the PPI network (**Figures 3A, B**), some genes were detected with a higher ranking score, such as upregulated genes in the EPC network and downregulated genes in the EcCentricity network (**Figures 3A, B**). Most genes were filtered if they were not simultaneously detected by Degree and other >4 algorithms, and only 31 genes (including 13 upregulated genes and 18 downregulated genes) were obtained as candidate hub genes associated with LUAD. Many hub genes were detected in multiple algorithms and simultaneously had higher degree scores, and most showed consistent scores in specific algorithms (**Figure 3**). These implied that candidate hub genes had higher confidence levels and might be the most influential proteins in PPI networks, further indicating that they might be crucial genes in tumorigenesis.

**Figure 4:**
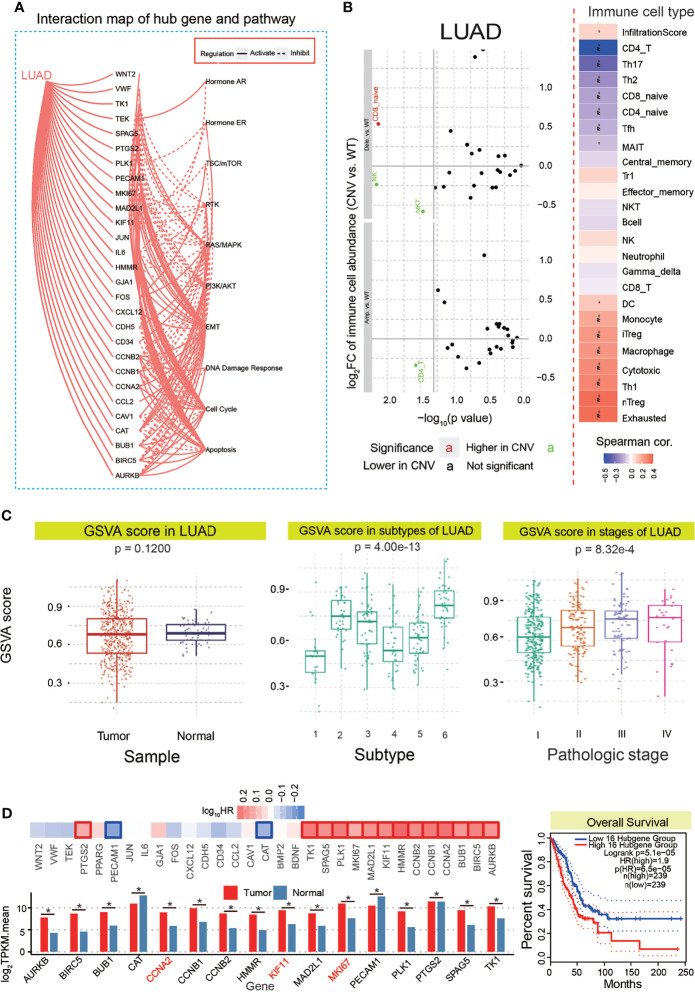
Functional analysis for 31 candidate hub genes and further screening. **(A)** Interaction map of 31 hub genes and pathways. **(B)** Potential roles of 31 hub genes in immune infiltrates. **(C)** Potential roles of 31 hub genes in LUAD, mainly including GSVA scores in tumor and normal samples, subtype, and stages in LUAD. **(D)** A final 16 genes are identified based on survival analysis, and all of these 16 genes showed abundant expression. The overall survival analysis is also presented. LUAD, lung adenocarcinoma; GSVA, gene set variation analysis. *p < 0.05.

To validate whether these candidate hub genes indeed had crucial roles in tumorigenesis, 31 genes were queried for the potential roles in biological pathways, apoptosis, cell cycle, DNA damage response, etc. These candidate hub genes were found to activate and inhibit some biological pathways (**Figures 4A** and **S3A**), implying their roles in relevant pathways that were crucial in the occurrence and development of cancer. Simultaneously, we have performed the analysis of the association between immune cells’ infiltrates and hub genes’ CNV levels. The results showed that CD4^+^ cells had a higher copy number variation (CNV) level in the hub gene CNV amplificated group than that in the wild-type group, and CD8_native cells had a significant CNV level in hub gene CNV deleted group compared with the wild-type group (**Figure 4B**). These genes did not show a significant difference between tumor and normal samples (p = 0.1200), but they showed significant differences among different subtypes of LUAD (p = 4.00e−13) and different stages of LUAD (p = 8.32e−4, **Figure 4C**). These varieties revealed that these screened genes were associated with subtypes and diverse stages. Moreover, these genes had positive or negative correlations with some drugs (**Figure S3B**). For example, trametinib was positively correlated with CCNA2, KIF11, MKI67, and MAD2L1. In June 2017, the Food and Drug Administration (FDA) approved trametinib plus dabrafenib for the treatment of BRAF V600E mutation-positive metastatic NSCLC patients. These showed the potential associations with anticancer drugs and roles as potential drug targets in future cancer treatment.

### Further Validation of Hub Genes and Relevant Non-Coding RNAs

To further survey and validate the hub genes associated with LUAD, their potential prognostic values were queried as an important index. A total of 16 genes were detected with significant prognostic values (**Figure 4D**), and all of them showed significantly deregulated expression patterns based on median expression values of tumor and normal samples. The overall survival curve of these genes showed that patients with lower expression had a higher survival probability than those with higher expression levels (**Figure 4D**). Accordingly, these candidate genes were identified as hub genes associated with LUAD, which were used to survey relevant miRNAs to explore the potential interactions among diverse RNAs, especially among mRNAs and ncRNAs. Interestingly, some of them were homologous genes in a specific gene family, including CCNA2, CCNB1, and CCNB2. Some of them, CCNA2, MKI67, and KIF11, were identified as cell cycle-related factors, implying their roles in the cell cycle pathway.

**Figure 5:**
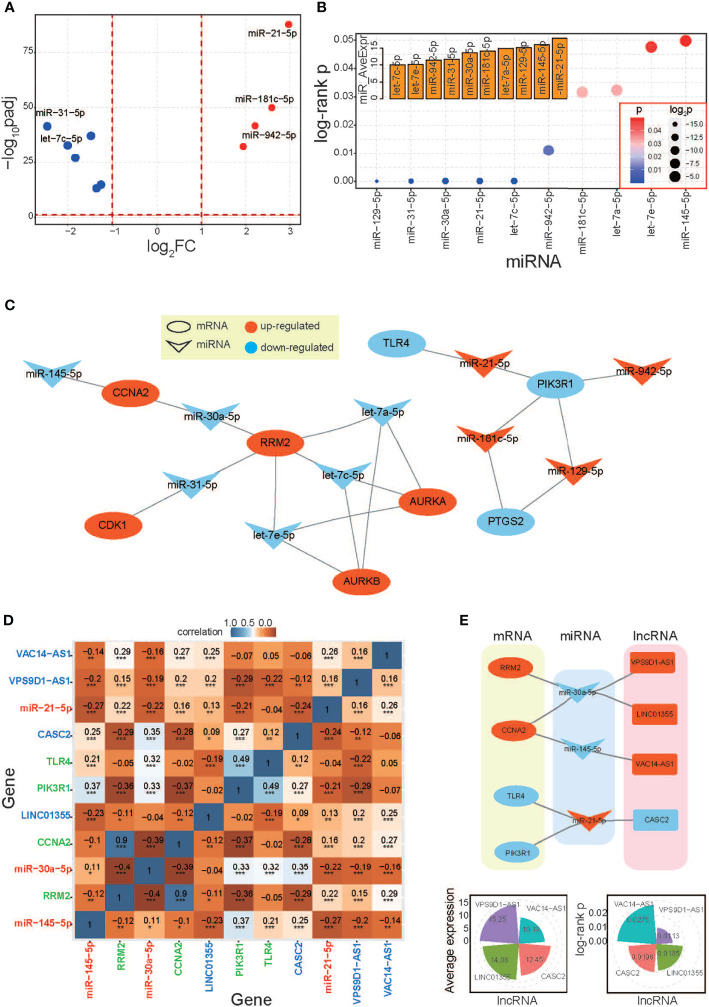
Screening the relevant RNAs based on hub genes. **(A)** A scatter plot shows distributions of log_2_FC and padj values of surveyed miRNAs, and all of these miRNAs have significant correlations with cancer prognosis. **(B)** The distributions of log-rank p-values of screened miRNAs (only significant results are presented) and the average expression levels of miRNAs are also presented. All of these involved miRNAs have abundant enrichment levels. **(C)** miRNA:mRNA interaction network based on their biological relationships. **(D)** Expression correlations among different RNAs. mRNAs, miRNAs, and lncRNAs are highlighted in different colors. **(E)** The relevant lncRNAs are further screened based on identified miRNAs, and the interactions networks among diverse RNAs are presented. The average expression of lncRNAs and log-rank p-values are also presented. LUAD, lung adenocarcinoma; GSVA, gene set variation analysis. *p < 0.05, **p < 0.01, ***p < 0.001.

A series of relevant miRNAs were surveyed based on the potential biological relationships with the 16 hub genes. Based on expression patterns and the significant correlations with cancer prognosis (log-rank p < 0.05), 10 miRNAs were obtained (**Figures 5A, B**). These miRNAs showed significant abnormal expression in LUAD, including 6 downregulated and 4 upregulated miRNAs, and all of them were detected with abundant enrichment levels. Of these, 3 of them were identified as homologous miRNAs, in let-7 gene family, and these miRNAs also had similar sequence, expression distributions, and biological roles. These miRNAs had opposite expression patterns with their target mRNAs (**Figure 5C**), implying their potential regulatory roles in the relevant mRNA expression process. Then, the primarily screened miRNAs were used to survey relevant lncRNAs based on their biological relationship. According to expression patterns and prognostic values, 2 lncRNAs as well as 4 mRNAs and 2 miRNAs were finally identified as candidate relevant RNAs, and most paired RNAs showed significant expression correlations (**Figures 5D** and **S3C**). These diverse RNAs showed potential regulatory relationships, and these obtained lncRNAs also had significant correlations with cancer prognosis and were detected with abundant enrichment levels (**Figure 5E**). Among these, both miR-145-5p and miR-30a-5p were identified regulators with 3 mRNAs and 1 lncRNA, respectively. These screened RNAs have been reported with important biological roles.

### Competing Endogenous RNA Construction and In-Depth Analysis

**Figure 6:**
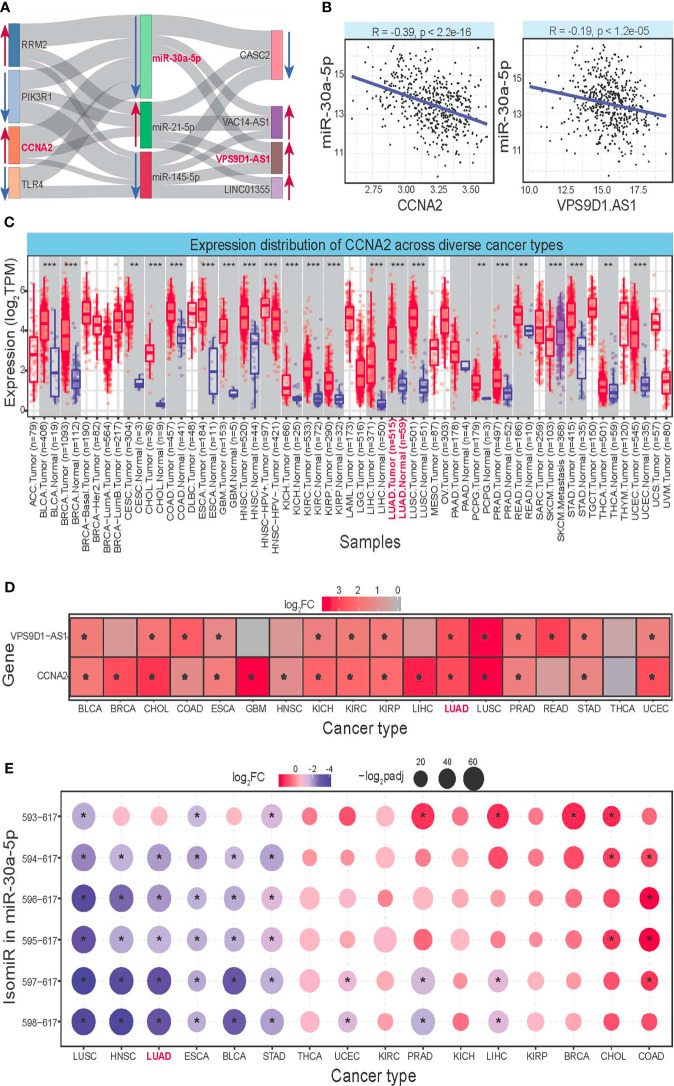
Construction of ceRNA network and further analysis for the involved RNAs. **(A)** Constructed ceRNA network based on obtained RNAs. The line shows the correlation between diverse RNAs, and the abnormal expression patterns are also highlighted using red arrows (upregulated) and blue arrows (downregulated). The red genes are further identified as potential crucial RNAs associated with LUAD. **(B)** The scatter plots show the negative expression correlation between miR-30a-5p and CCNA2 and VPS9D1-AS1. **(C)** Expression distributions of CCNA2, MKI67, and KIF11 in tumor and normal samples across diverse cancer types. *p < 0.05, **p < 0.01, ***p < 0.001. **(D)** Deregulated expression patterns for VPS9D1-AS1 based on log_2_FC values. * indicates significant abnormal expression (log_2_FC > 1.2, padj < 0.05). **(E)** Expression distributions of the multiple isomiRs in miR-30a-5p locus across diverse cancer types, and only the dominantly expressed isomiRs are presented here (the top 6 isomiRs). These isomiRs are presented using the detailed location, and 593–617 indicate hg38:chr6:71403593–71403617:–. ceRNA, competing endogenous RNA; LUAD, lung adenocarcinoma.

A total of 8 diverse RNAs were used to construct a ceRNA network based on their expression correlations (**Figure 6A**), showing their potential interactions across different RNAs, especially among ncRNA and mRNAs. LncRNA may control mRNA expression *via* binding to the regulator of mRNA and miRNA, and the complex interactions might further complicate the coding–non-coding RNA regulatory network. Based on involving RNAs in the ceRNA network, further analysis was performed to verify their regulatory interaction, mainly including expression level, expression correlation, and survival analysis. Finally, the 5 RNAs, including CCNA2, MKI67, KIF11, miR-30a-5p, and VPS9D1-AS1, were further identified as candidate crucial RNAs associated with cancer. A significant expression correlation could be found between miRNA and its relevant mRNA and lncRNA (**Figure 6B**), and an in-depth analysis of the three RNAs was performed to verify their potential biological roles.

To understand the potential roles of surveyed RNAs in other cancer types, a pan-cancer analysis was performed to discuss their expression patterns. Involved genes (CCNA2, MKI67, and KIF11) were found with abundant expression levels in many tissues, and they showed a significantly upregulated expression pattern in many cancer types (**Figure 6C**). Simultaneously, lncRNA VPS9D1-AS1 also showed a significant overexpression pattern in many cancer types (**Figure 6D**), and the consistent expression trends implied their competition binding with miR-30a-5p. Moreover, although miR-30a-5p was identified as a crucial miRNA, it is not a single miRNA but a series of multiple isomiRs. Then, based on dominantly expressed isomiRs, 6 abundant isomiR were selected, and they showed diverse expression patterns than mRNAs and lncRNAs (**Figure 6E**). The dynamic expression of isomiRs implied their flexible regulatory expression, which may contribute to specific biological pathways in different tissues based on their broad-spectrum target RNAs. Further, these 6 dominant isomiRs were found with the consistent 5′ ends and seed sequences (nucleotides 2–8) that were binding sites with target RNAs, and they were only involved differently in the 3′ ends and diverse expression patterns. It is unclear whether the length difference would influence stability or regulation efficiency, but most of them were found with unexpected enrichment levels that ensured their biological function. These isomiRs with the same seed sequences have diverse length and expression levels, which would further complicate the interaction network among coding–non-coding RNA regulatory networks.

**Figure 7:**
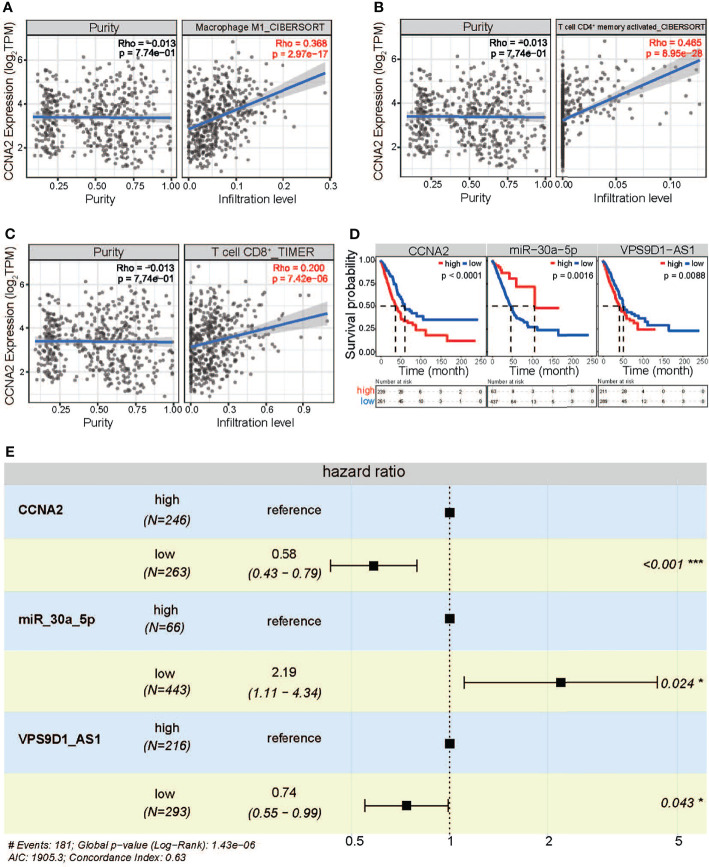
In-depth analysis of screened RNAs. **(A)** The expression correlation of CCNA2 in Macrophage. **(B)** The expression correlation of CCNA2 in T cell CD4+ memory activated. **(C)** The expression correlation of CCNA2 in T cell CD8+. **(D)** The survival analysis for the three RNAs. **(E)** Hazard ratio analysis based on forest plot. *p < 0.05, ***p < 0.001.

Furthermore, the crucial genes, CCNA2, MKI67, and KIF11, were further queried for their roles in immune infiltration in LUAD. In different immune cell types, all of them showed a significant positive correlation with immune infiltration (**Figures 7A–C**). These results showed that a higher expression level of CCNA2, MKI67, and KIF11 might lead to higher infiltration levels, implying their roles in immune infiltration, a key step in the pathological process of cancer.

### Potential Prognostic Marker *via* RNA Cross-Talk

As cancer-associated crucial RNAs, the 5 screened RNAs showed a significant difference between groups with high and low expression, and patients with higher expression of mRNAs and lncRNA had a poorer prognosis than those with lower expressions (p < 0.0001, p = 0.00014, p < 0.0001, and p = 0.0088, **Figure 7D**). However, patients with lower expression of miR-30a-5p had a poorer prognosis than those with higher expressions (p = 0.0016). Their prognostic values were also verified by analysis of hazard ratio (the global log-rank p = 1.43e−06, **Figure 7E**). These results significantly showed that different RNAs, CCNA2/MKI67/KIF11:miR-30a-5p:VPS9D1-AS1 axis-related cell cycle, could be a potential prognostic marker *via* RNA cross-talk, especially for the cross-talks among ncRNAs and mRNAs (**Figure S3D**).

Furthermore, CCNA2 and KIF11 were identified as core essential genes according to the common data of Hart et al. (51), Blomen et al. (52), and Wang et al. (53). CCNA2 contributed to the cell cycle pathway, and it also had a role in the hallmarks of cancer in reprogramming energy metabolism. These contributions implied their key role in the occurrence and development of LUAD, even in cancer diagnosis and prognosis. The interactions with CCNA2, MKI67, and KIF11, particularly for the small and long ncRNAs, may have great importance as potential drug targets based on their contributions in multiple biological pathways (**Figures S3A, B**).

## Discussion

Based on the potential interactions or cross-talks among different RNAs, it is quite necessary to perform an integrative analysis to survey the relevant RNAs as a potential prognostic marker. Due to the fact of being the leading cause of cancer-related death, lung cancer has been widely of concern, and it is urgent to obtain prognostic markers with higher sensitivity that will largely contribute to adjusting drugs and cancer treatment, especially in precision medicine.

Herein, based on an integrative analysis of diverse RNAs from different datasets, CCNA2/MKI67/KIF11:miR-30a-5p:VPS9D1-AS1 axis-related cell cycle is identified as a potential prognostic marker *via* constructing a ceRNA network and in-depth analysis, and all of them are characterized as crucial RNAs in the occurrence and development of LUAD. Of the three mRNAs, CCNA2 has been studied because of its role in cancer, including its prognostic value in breast cancer (54–56), colorectal cancer (57), pancreatic cancer (58), LUAD (59), gastric cancer (60), bladder cancer (61), etc. MKI67, a marker gene in the cell cycle, also has been reported with prognostic value in NSCLC (62) and breast cancer (63). Furthermore, the prognostic value of KIF11 has been reported in oral cancer (64) and colorectal cancer (65).

Our analysis shows that CCNA2 is an important gene in the cell cycle, and it is significantly upregulated in many cancer types. The disorder of CCNA2 contributes to multiple cancers, implying its potential role in cancer diagnosis and prognosis. Tanshinone IIA can significantly downregulate the expression of the CCNA2–CDK2 complex and suppress the progression of LUAD by inducing cell apoptosis and arresting the cell cycle (66). One of its regulators, miR-30a-5p, also has been widely of concern as an important miRNA, especially for its role *via* cross-talk with other RNAs in some pathways in different cancers (67–69). The overexpression of another ncRNA, lncRNA VPS9D1-AS1, a potential prognostic marker, can be used to predict poor prognosis in NSCLC (70), and its role in cancer has been validated (71, 72). All of these RNAs have been validated with roles in tumorigenesis, and this axis may be a proper marker to predict cancer progression.

Meanwhile, based on the widespread phenomenon of isomiRs occurring in the miRNA locus, the screened crucial miR-30a-5p is also further analyzed at multiple isomiR levels. A series of multiple isomiRs can be detected, and dominantly expressed isomiRs are also unexpectedly enriched, which may ensure their regulatory roles. Although these isomiRs are not involved in causing the differences of 5′ ends and seed shifting events, their expression and length difference still provide a possibility to perturb the original coding–non-coding RNA regulatory network. The main reason may possibly be derived from these isomiRs with expression and sequence heterogeneities, but it is unclear whether these isomiRs may competitively bind to target RNA (mRNA and lncRNA). If the 5′ ends are involved differently, the novel seed sequences will be found, which may lead to some novel targets simultaneously losing some targets. It is quite necessary to perform analysis from the multiple isomiR levels despite many studies only focusing on the traditional/classical miRNAs. The small ncRNAs largely contribute to the complex cross-talks among diverse RNAs, especially in coding–non-coding RNA regulatory network, which is more complex than we thought because of the phenomenon of isomiRs in the miRNA locus.

Taken together, based on the potential cross-talks among diverse RNAs, this study finally screened and identified CCNA2/miR-30a-5p/VPS9D1-AS1 axis as a potential prognostic marker in LUAD. All of the relevant RNAs have been widely studied with roles in the occurrence and development of cancers, indicating their crucial roles in tumorigenesis, especially for association with cell cycle *via* direct or indirect contribution. Further study should focus on their values as a potential therapeutic target for cancer treatment. Our findings will provide insight into cross-talks among diverse RNAs, especially from the unique perspective of multiple isomiRs from a given miRNA gene locus, which will enrich our understanding of mRNA–ncRNA interactions in coding–non-coding RNA regulatory network in tumorigenesis.

## Data Availability Statement

The original contributions presented in the study are included in the article/**Supplementary Material**. Further inquiries can be directed to the corresponding author.

## Author Contributions

YY and LG designed this study. YY, SZ, and LG participated in the data analysis. LG and YY wrote the manuscript. All authors read and accepted the final version.

## Funding

This work was supported by the National Natural Science Foundation of China (Nos. 61771251 and 62171236), the key project of social development in Jiangsu Province (No. BE2016773), and the National Natural Science Foundation of Jiangsu (No. BK20171443) and sponsored by NUPTSF (No. NY220041) and the Qinglan Project in Jiangsu Province.

## Conflict of Interest

The authors declare that the research was conducted in the absence of any commercial or financial relationships that could be construed as a potential conflict of interest.

## Publisher’s Note

All claims expressed in this article are solely those of the authors and do not necessarily represent those of their affiliated organizations, or those of the publisher, the editors and the reviewers. Any product that may be evaluated in this article, or claim that may be made by its manufacturer, is not guaranteed or endorsed by the publisher.

## Abbreviations

ACC, adrenocortical carcinoma; BLCA, bladder urothelial carcinoma; BRCA, breast invasive carcinoma; CESC, Cervical squamous cell carcinoma and endocervical adenocarcinoma; CHOL, cholangiocarcinoma; COAD, colon adenocarcinoma; DLBC, lymphoid neoplasm diffuse large B-cell lymphoma; ESCA, esophageal carcinoma; GBM, glioblastoma multiforme; HNSC, head and neck squamous cell carcinoma; KICH, kidney chromophobe; KIRC, Kidney renal clear cell carcinoma; KIRP, kidney renal papillary cell carcinoma; LAML, acute myeloid leukemia; LIHC, liver hepatocellular carcinoma; LGG, brain Lower grade glioma; LUAD, lung adenocarcinoma; LUSC, lung squamous cell carcinoma; MESO, Mesothelioma; OV, ovarian serous cystadenocarcinoma; PAAD, pancreatic adenocarcinoma; PCPG, pheochromocytoma and paraganglioma; PRAD, prostate adenocarcinoma; READ, rectum adenocarcinoma; SARC, sarcoma; SKCM, skin cutaneous melanoma; STAD, stomach adenocarcinoma; TGCT, testicular germ cell tumors; THCA, thyroid carcinoma; THYM, thymoma; TSG, tumor suppressor gene; UCEC, uterine corpus endometrial carcinoma; UCS, uterine carcinosarcoma; UVM, uveal melanoma.
