# Supplementary material for: Characterization of Cell Cycle-Related Competing Endogenous RNAs Using Robust Rank Aggregation as Prognostic Biomarker in Lung Adenocarcinoma
Source: Front Oncol. 2022 Feb 3;12:807367. doi: 10.3389/fonc.2022.807367 (PMC8853726; doi:10.3389/fonc.2022.807367)
Supplement: Supplementary file 1 [file DataSheet_1.pdf]

Supplementary materials

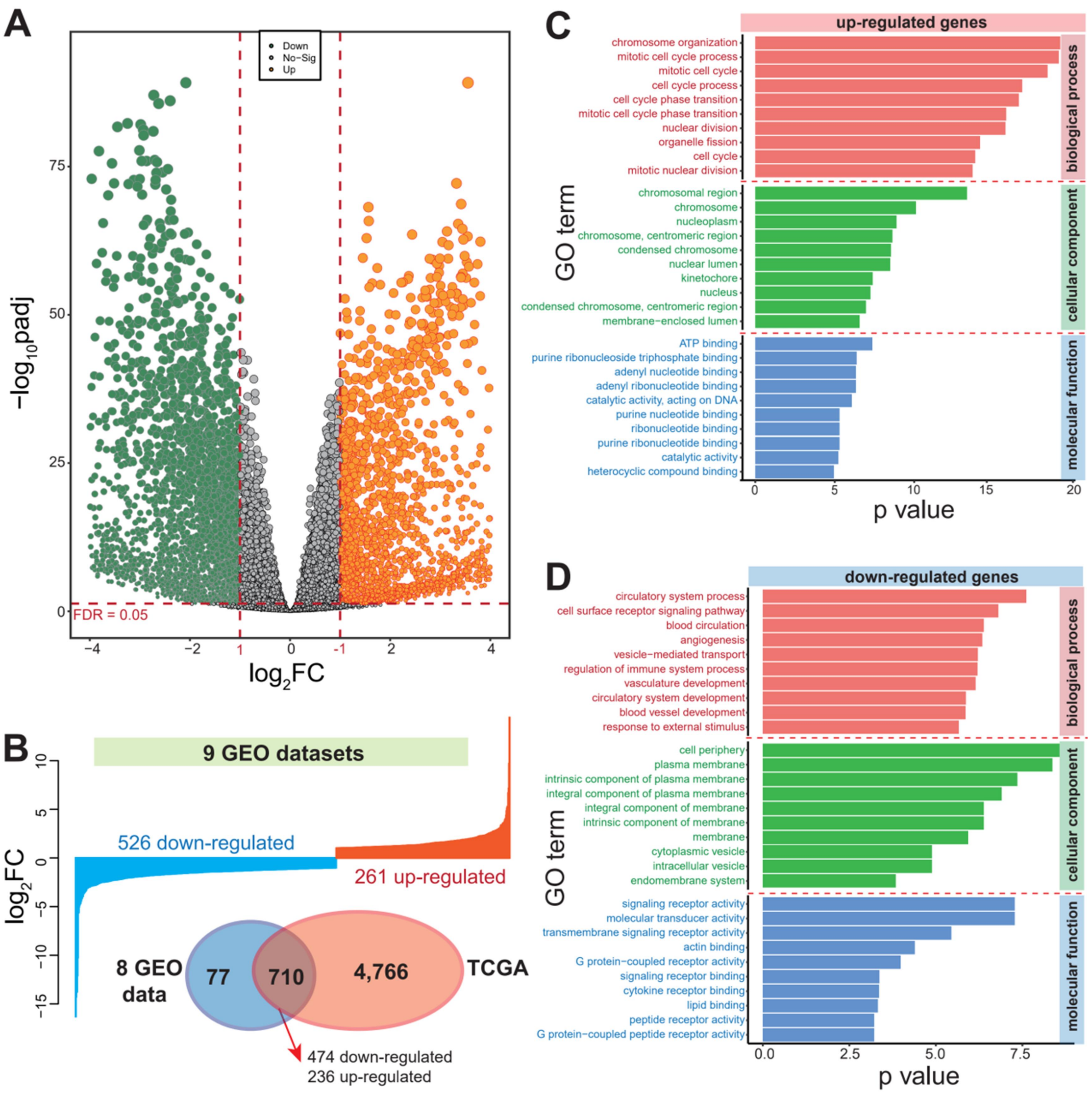

Figure S1. Screening consistent genes and functional analysis.

A. The distributions of RRA scores via integrative analysis of 9 GEO datasets. Venn distribution shows that a total of 710 consistent deregulated genes are collected through GEO and TCGA datasets.

B. A scatter plot shows the expression distributions of all the relevant genes in TCGA dataset.

C. Significantly enriched GO terms based on up-regulated screened genes. BP indicates biological process, CC indicates cellular component, and MF indicates molecular function.

D. Significantly enriched GO terms based on down-regulated screened genes.

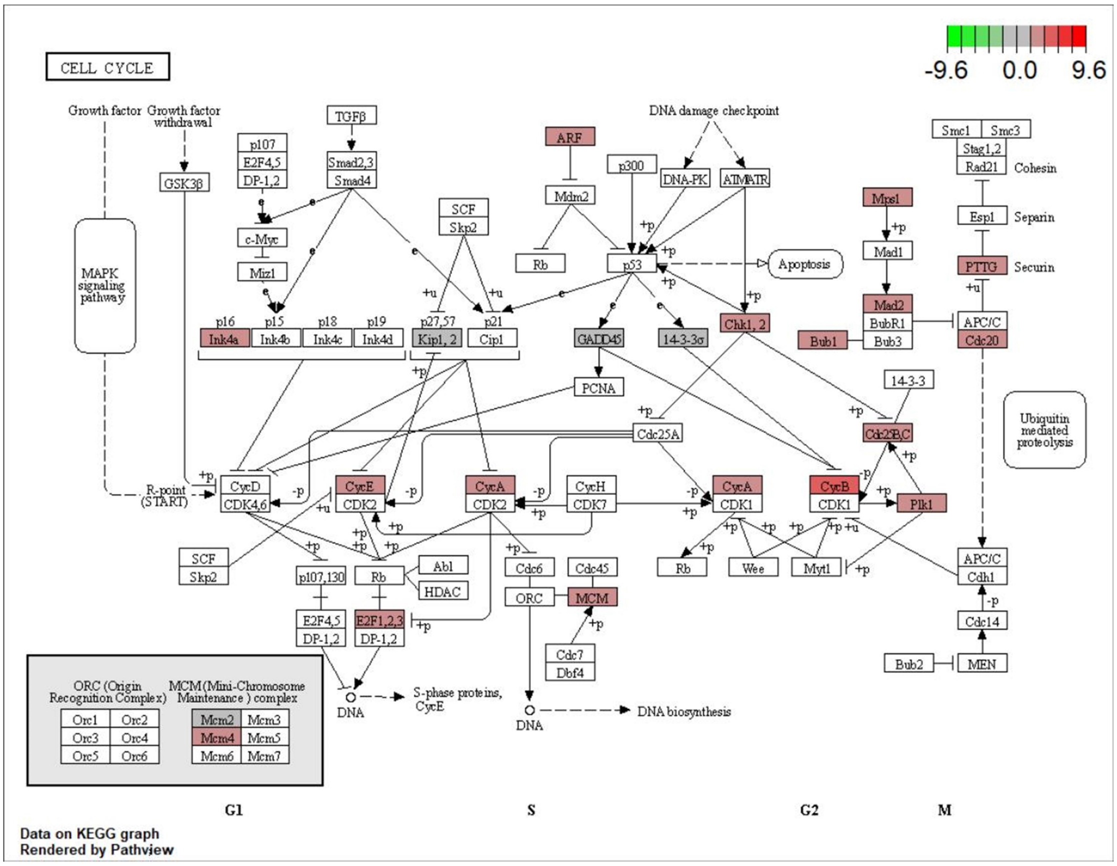

Figure S2. Contributed cell cycle pathway by analyzing primarily screened 710 genes.

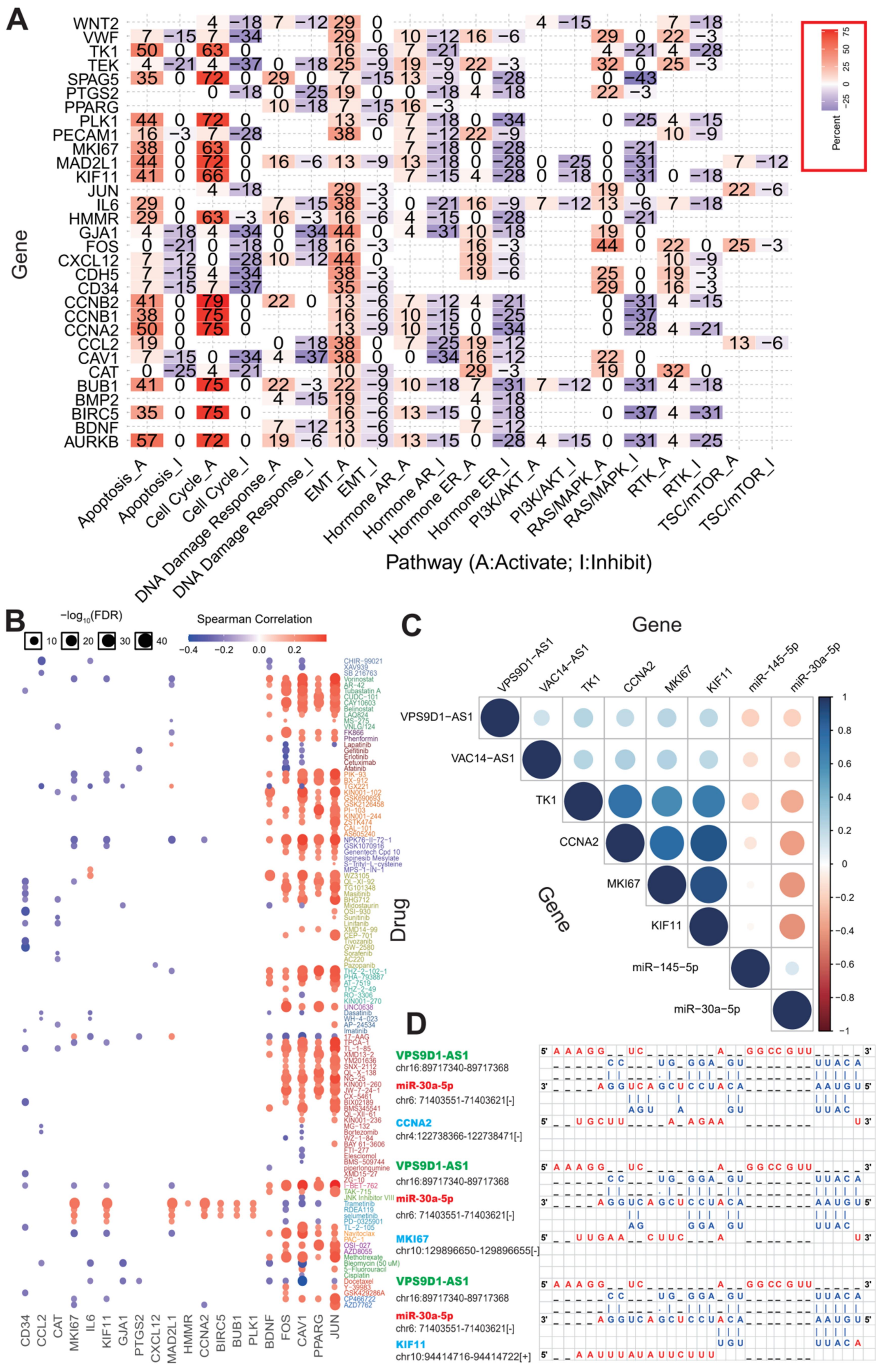

Figure S3. Functional analysis of the screened 31 hub genes.

A. All of these 31 genes are detected certain roles in activating and inhibiting pathways.

B. The correlations of these 31 hub genes with drug sensitivity.

C. The expression correlations between paired genes.

D. The binding sites between different RNAs.

Table S1. The gene expression data in LUAD used in the study.

Datasets	Tumor	Normal	Total	Platforms	PMID number
GSE31210	226	20	246	GPL570	PMID: 23028479
GSE118370	6	6	12	GPL570	PMID: 30545439
GSE75037	83	83	166	GPL6884	PMID: 27354471
GSE32863	58	58	116	GPL6884	PMID:22613842
GSE85716	6	6	12	GPL19612	PMID: 29127420
GSE85841	8	8	16	GPL20115	PMID: 28178989
GSE63459	33	32	65	GPL6883	PMID: 26134223
GSE130779	8	8	16	GPL20115	PMID: 32869486
GSE148036	5	5	10	GPL21290	PMID: 33097805
TCGA-LUAD	483	59	542	IlluminaHiSeq	
Total	916	285	1201		
